# Self-Injurious Behavior in Child and Adolescent Psychiatry Inpatient Units: Actual Aspects of the Complex Care Provision

**DOI:** 10.15388/Amed.2024.31.2.7

**Published:** 2024-12-04

**Authors:** Sigita Lesinskienė, Mariam Afrahi, Kamilė Pociūtė

**Affiliations:** 1 Clinic of Psychiatry, Institute of Clinical Medicine, Faculty of Medicine, Vilnius University, Vilnius, Lithuania; 2 Faculty of Medicine, Vilnius University, Vilnius, Lithuania

**Keywords:** self-harm, inpatient units, clinical aspects, children, adolescents, Raktažodžiai: savižala, stacionarai, klinikiniai aspektai, vaikai, paaugliai

## Abstract

**Background:**

Nonsuicidal self-injurious behavior in children and adolescents is a major concern that requires mental health professionals’ attention. The aim of this study is to analyze clinical care aspects of children and adolescents who self-harm in psychiatric hospitals.

**Materials and methods:**

In 2023, 30 various specialists from five different child and adolescent psychiatric units in Lithuania were interviewed. The survey used a semistructured interview consisting of twelve questions related to complex clinical care methods of children and adolescents who self-harm. The interview responses were summarized and grouped into 5 categories: assessment and monitoring, methods and consequences of self-injury, safety measures, prevention and treatment, insights from staff.

**Results:**

Self-injurious behavior in psychiatric inpatient settings was managed through risk assessment, monitoring, communication, medication, counselling, removal of sharp objects, patient allocation, and a several of other methods such as safety contracts, rewards or alternate pain-inducing or self-harm mimicking stimuli. Despite the hospital’s safety procedures patients frequently devised alternate methods to self-harm, such as hitting and scratching themselves and using nonspecific materials.

**Conclusions:**

The management of self-harm in children and adolescents psychiatric settings remains insufficient. Further research is needed to explore alternative ways of managing self-injurious behavior in child and adolescent psychiatric hospitals.

## Abstract (it)

**Įvadas:**

Vaikų ir paauglių save žalojantis elegesys be savižudybės ketinimo yra reikšminga problema, kuriai reikia psichikos sveikatos specialistų dėmesio. Šio tyrimo tikslas – išanalizuoti klinikinės praktikos aspektus teikiant stacionarinę pagalbą save žalojantiems vaikams ir paaugliams.

**Metodai:**

2023 metais apklausta 30 įvairių specialistų iš penkių skirtingų vaikų ir paauglių psichiatrijos stacionarų, veikiančių Lietuvoje. Apklausai naudotas pusiau struktūruotas interviu, kurį sudarė dvylika klausimų, susijusių su taikomais kompleksinės pagalbos metodais vaikams ir paaugliams, kurie žalojasi. Interviu atsakymai apibendrinti ir sugrupuoti į 5 kategorijas: vertinimas ir stebėsena, savęs žalojimo būdai ir pasek-mės, saugumo priemonės, prevencija ir gydymas, personalo įžvalgos.

**Rezultatai:**

Save žalojantis elgeys psichiatrijos stacionaruose buvo valdomas vertinant riziką, vykdant stebėseną, komunikuojant, taikant medikamentinį gydymą, konsultuojant, pašalinant aštrius daiktus, išskirstant pacientus bei naudojant kitus metodus, kaip antai – saugumo kontraktai, apdovanojimai ar pakaitiniai skausmą sukeliantys ar savižalą imituojantys stimulai. Nepaisant stacionarų personalo pastangų užtikrinti pacientų saugumą, pacientai dažnai naudojo alternatyvius savižalos metodus, kaip trankymas, savęs draskymas ir nespecifinių priemonių naudojimas.

**Išvados:**

Savižalos valdymas vaikų ir paauglių psichiatrijos stacionaruose gali būti nepakankamai efektyvus. Reikėtų tolesnių tyrimų šioje srityje, siekiant išsiaiškinti veiksmingus savižalos valdymo būdus vaikų ir paauglių psichiatrijos stacionaruose.

## Introduction

Nonsuicidal self-injury (NSSI) is a common issue among hospitalized children and adolescents. NSSI is characterized by intentional self-harm without suicidal intent, typically starting between the ages of 12 and 14 [1], with a peak during 14–15 years and decline during late adolescence [2]. Self-harm is found to have increased several-fold over decades in the adolescents’ population [3]. The lifetime prevalence among the general adolescent population is 13–27.6% [4, 5], with higher rates in clinical settings [6].

The pathogenesis of NSSI is multifactorial and not clearly understood. The authors of one study suggest that self-harm might be seen as a compulsive rather than impulsive disorder, providing a novel perspective on this behavior [7]. Females may be at higher risk, and gender differences exist for self-injury methods. Females report a greater frequency of injuries to their arms and legs, while males report more injuries to the face, chest, and genital areas. Additionally, females engage in more cutting and scratching, whereas men report a higher prevalence of burning and hitting-type behaviors [8]. Psychological distress is a consistent factor for triggering self-injurious behaviors. Factors that can induce distress, such as a sense of isolation, feeling disconnected from others, and exposure to self-harm, can trigger the urge to self-harm among young people [9]. A meta-analysis found that mental disorders, low health literacy, problem behaviors, bullying, adverse childhood experiences, and female gender are risk factors for NSSI [10]. Another meta-analysis identified that prior NSSI history, cluster B personality disorders, and hopelessness are all significant risk factors [11].

The presence of NSSI is associated with increased inclination and capability for suicide and is one of the strongest predictors of future suicide attempts [12, 13]. Earlier onset of self-harming behavior, specifically before the age of 12, has been linked to higher self-harm frequency, a wider variety of self-harming methods, and higher rates of hospitalization [14]. Moreover, inpatients exhibited a considerably earlier age of onset for NSSI compared to the outpatient sample [13]. Violent self-harm, such as hanging, strangulation, drowning, jumping, or gassing, necessitating medical hospitalization, may indicate a significantly increased risk of future suicide among adolescents of both genders and young adult women [15].

There is no gold standard treatment for NSSI [16]. Usual inpatient treatment of self-harm often requires individual staffing and/or constant monitoring, when required medication, and the removal of all sharp objects from the environment. Clarity on the appropriate treatment and management of NSSI remains a challenge [17]. While high NSSI prevalence persists in inpatient units, there is scant evidence that current inpatient treatments effectively deter such behavior [18]. Given the high prevalence of self-injurious behavior among children and adolescents, understanding how healthcare providers evaluate and manage this behavior is critical.

This article aims to investigate NSSI management approaches for self-injurious behaviors in inpatient settings, including self-harm assessment, safety measures, prevention, and treatment, as well as staff’s experiences and their perceptions of the effectiveness of approaches to reduce self-harm. We hypothesize that the most used interventions to reduce self-harm in inpatient settings involve the removal or modification of self-harm tools and that practitioners might assume that such methods are not enough to reduce self-harm. The study was obtained through an interview process with personnel from five different children and adolescents’ psychiatric units in Lithuania. The findings are discussed along with existing literature, with the hope that this will contribute to the development of future research and effective interventions to address self-harm in children and adolescents.

## Materials and Methods

A survey was conducted in 2023 based on semistructured interviews with personnel in children’s psychiatric departments in Lithuania, focusing on the topic of self-injurious behavior. The participants were recruited from five different psychiatric inpatient units across Lithuania. The participants were selected based on their availability and willingness to participate in the study. A total of 30 participants were interviewed. The participants included psychiatrists, psychologists, nurses, social workers, occupational therapists, teachers, and physiotherapists. Inpatient workers had contact with patients aged 6 to 17 with various mental health conditions, including depression, anxiety, eating disorders, conduct disorder, obsessive-compulsive disorder, autism, attention-deficit/hyperactivity disorder, psychosis, and backgrounds of trauma and/or abuse.

Data was gathered in person through interviews using a semistructured questionnaire with 12 questions:

1. What is your role or position?
2. Have you encountered self-harming patients or behaviors while in the hospital?
3. What methods or self-harm have you observed or heard of in unit patients?
4. What are the consequences of self-harm?
5. How do you identify at-risk patients?
6. Is the patient’s body and/or belongings examined? Who is responsible for this, and what warrants it?
7. How do you approach a potentially affected child about self-harm?
8. How is the incidence of self-harm addressed (e.g., protocol, interventions)?
9. Are there any preventive or other measures in place to address self-harm?
10. How do employees collaborate to report and respond to self-harm?
11. How do you feel about patients’ self-harming behavior?
12. Do you have any suggestions for improving self-harm management in the department?

Additional follow-up questions were presented to explore a topic further or provide clarification. When necessary, the order of the questions was changed to provide continuity. Interview responses were analyzed, and the results were grouped into 5 categories: assessment and monitoring, methods and consequences of self-injury, safety measures, prevention and treatment, and insights from staff. We have summarized the results in Table 1.

**Table 1:** Summary of the results

Assessment and monitoring	Methods and consequences of self-injury	Safety measures	Prevention and treatment	Insights from staff
Conducting interviews and questionnaires by psychiatrists and psychologists Reporting incidents to other specialists immediately or at a scheduled time Marking higher-risk patients on the board Monitoring higher-risk patients more frequently Observing patients during mealtimes Performing night-time check-ins Leaving ward doors open Locating wards closer to nurses Using wards with glass walls	Cutting with pins, wires, razors, broken glass, knives, etc. Hitting a wall, scratching with nails, rubbing with rubber, etc. Using less common methods of self-harm, such as swallowing a spoon, drinking abnormally large amounts of water, etc. Engaging in self-harm alone or as a group Learning to self-harm from other patients More common following admission and before discharge Meetings with parents could trigger self-harm Most cases do not necessitate medical treatment	Restricting the use of certain utensils or changing materials to plastic Placing patients with similar problems in different rooms Checking the patients’ belongings Permitting personal items such as necklaces, earrings, etc. Locking doors and removing doorknobs Accounting for sharp objects in places such as classrooms, offices, etc. Video surveillance No-harm contract Crisis plan Reward system	Observation Performing risk assessments throughout the stay Communicating between staff members Addressing self-harm behavior in therapy sessions Providing additional sessions if needed Replacing self-harming behaviors (ice, cold water, spices, lemon, etc.) Medication Restricting smartphone use Including social workers No formal training for hospital workers is available	The management of self-harm in inpatient settings may be insufficient; the focus should be on emotional regulation, positive reinforcement, and devising alternative coping strategies It is important not to blame the patient More training and support for mental health specialists is needed Dealing with patients who self-harm can lead professionals to experience neutral to negative emotions. Working with adolescents can lead to heightened concerns for safety Specific patient groups should be treated in specific departments

## Results

### Assessment and monitoring

Upon admission, the assessment of self-harm risk and suicidality is standard practice. Nonsuicidal self-injury was assessed upon admission through interviews, questionnaires, and patients’ skin examinations. Clinicians took a proactive approach to self-harm by asking about it directly during sessions. Furthermore, patients would occasionally disclose self-harm behaviors independently, especially if they were participating in group activities involving self-harm or observed such behaviors in others. To enhance patient safety and effectively monitor those at risk, one hospital implemented a system where all inpatients were listed on a whiteboard in the staff area. Those with a high risk of suicide were marked with an “S.” Additionally, a separate column on the board listed specific behaviors to watch for according to each patient’s individualized treatment plan. These behaviors included eating patterns (for individuals with eating disorders), sleep patterns, and tendencies toward self-harm, among other factors. A nurse was often the initial responder to the event of self-harm, assessing the seriousness of the problem and providing necessary medical treatment. Subsequently, the incidents were reported to other relevant staff members and clinicians during scheduled reporting sessions or, if necessary, immediately. During waking and sleeping hours, nurses attempted to closely monitor patients. While patients were permitted to sleep with their doors closed, one department frequently left the doors to patients’ rooms open until patients fell asleep to ensure their safety. The frequency of night-time check-ins varied across hospitals, although high-risk patients were monitored more frequently. Nurses also observed patients during mealtime, ensuring a safe environment and preventing any incidents. One nurse described developing an “intuition” when it came to identifying at-risk patients. They emphasized that abrupt changes in a patient’s demeanor, such as increased withdrawal, were cause for concern and prompted proactive engagement. In such cases, the nurse would approach the individual to check in on them and, if necessary, initiate a discussion regarding potential self-harming behaviors. The nurses’ station was almost always located in the center of the department, which facilitated effective patient monitoring. Most hospitals strategically placed high-risk patients, including those at risk of suicide, closer to the nurses’ station. One hospital featured a room adjacent to the nurses’ station with glass walls (and curtains for privacy if needed) to allow for continuous observation. Patients who were at risk of self-harm were subjected to increased monitoring, with nurses performing more frequent checks during the night and throughout the day as needed. A nurse shared a telling anecdote, saying how she could try to have a conversation with a group of children about self-harm, unaware that one child might be engaging in such behaviors “behind her back,” unbeknownst to her. This narrative underscores the inherent challenges of appropriately monitoring all patients at all times.

### Methods and consequences of self-injury

It was agreed that individuals who are determined to harm themselves may find alternative methods to do so even if safety measures are in place and tools are removed. These methods included hitting themselves against a wall, punching a wall, scratching themselves, or using other nonspecific items. Patients frequently injured themselves using materials found in the hospital or brought in from outside, such as pins, wires, razors, and broken glass. There were isolated reports of broken Christmas ornaments and shattered phone screen protectors being used as cutting instruments. Mugs have also been destroyed and used as implements of self-harm. In one example, a patient gravely injured themselves with a knife during mealtime, and another swallowed a spoon. During COVID, the use of medical masks became prevalent, and patients began harming themselves with metal strips from the masks. Erasers were also rubbed on the skin to cause a burn. Patients would also scratch themselves with their nails or hit themselves against a surface. One patient drank excessive amounts of water, placing them at risk of water intoxication. Patients had also strangled themselves with clothing, such as a bra, or cut themselves with the steel support of the bra. Spiral notebook metal had also been used for self-harm. Patients could also create tools from materials and food brought in for them by visitors. Patients were also said to have coordinated the delivery of items to the department, either for themselves or for friends who were admitted or were soon to be admitted. Self-harm often happened alone, although it could also be a group activity, with some injuring themselves in private, while others do so in front of other patients and even personnel. In one case, someone had smuggled a cutter into the department and given it to three other people with a history of self-harm so they could all harm themselves together. While self-harm was frequently thought to be an existing behavior that persisted in the department, staff members mentioned instances where it may have emerged as a new behavior, such as when it was learned from others. These incidents included self-cutting and rubbing their skin with rubber. Arms were among the commonly injured areas, but skin on thighs and chest areas was also harmed. Several factors were said to influence the timing of self-harm during the hospitalization period. While self-harm could occur at any stage, such as the beginning, middle, or end, it was thought that such incidents were more common following admission and before discharge, especially if patients were reluctant to leave. Patient meetings and conversations with parents were often seen as triggering events for self-harm. Most physical consequences following a self-harm incident were described as superficial and not needing medical treatment (e.g., surgical dressing). No cases of suicide were reported in any of the hospitals. However, there were a few cases of major self-injury that required surgical or more intensive medical treatment.

### Safety measures

Every instance of self-harm appeared to be a teaching opportunity. Hospitals, for example, where mugs and utensils were used as means of self-harm either changed the materials to plastic and/or restricted or prohibited the use of certain utensils (such as knives and forks). All hospitals monitored the patients during mealtime, making sure there were no incidents. Moreover, patients with similar problems, such as those with eating disorders or suicide risk, were placed in different rooms to prevent patients from encouraging such behavior in each other and discussing strategies. During admission, nurses almost frequently checked the patients’ belongings. This was especially true if the patient was suspected of carrying an item that could be used for self-harm (for example, wearing baggy clothes or having a history of self-harm). Patients often devised creative methods to conceal materials that could be used for self-harm. For example, they could stitch hidden pockets into their clothing to conceal items, or they might conceal something in their bra or hair. Using bras with metal support was specifically prohibited, and if metal sections were discovered, they were either removed or parents were encouraged to bring metal-free alternatives, such as athletic bras. Some personal items, such as necklaces and earrings, were permitted given that they were not used for self-harm. Permission to have such personal items also depended on the individual’s risk of self-harm. Additionally, visitors were instructed not to bring materials that could be used for self-harm, and nurses would inspect items brought in by parents to ensure they could not be used for self-harm. The doors leading outside were always locked, and patients were not permitted to leave the department at their leisure. Within the department, offices, and spaces that were not redeemed freely accessible were also kept locked to ensure patient safety. One department had removed the doorknobs to stop patients from entering forbidden areas or leaving without permission, with staff only having access to a universal doorknob. When patients were in places with potential access to sharp objects, such as classrooms, offices, and occupational therapy settings, it was made sure that such items were clearly accounted for, and patients were never left in the room alone. Doors often had a glass window that would allow staff to check on occurrences inside the room. One hospital implemented live video surveillance in all patient rooms for monitoring. A person in charge reviewed the footage in the nurses’ station. Another hospital also had video surveillance, but the footage was limited to hallways visible only to general hospital security. One hospital required patients to sign a no-harm contract, agreeing not to injure themselves while in the hospital. Another department advised patients not to engage in this practice, but no official agreement was in place. During the hospitalization period, at least two hospitals worked with the patients to develop a crisis plan that included emergency contact in the event of a crisis, warning signs that typically precede or accompany self-harming urges, coping strategies that can be used as an alternative to self-harm, safety measures, and distractions to reduce distress. Patients often filled out these forms with the assistance of social workers. Another department instituted a reward system for a consistent record of nonharm and good behavior. This was a ladder system, with higher levels of advancement resulting in more lucrative rewards, such as a field trip. Although there were no direct consequences for self-harm, certain actions could potentially impede progress, affecting the track record.

### Prevention and treatment

Approaches for reducing self-harm incidents included observation, risk assessment (carried out during admission and throughout the stay), and communication between staff members regarding patient behavior. There appeared to be no formal training provided for hospital workers on dealing with self-harm. Thus, specific therapies to reduce self-harm have not been applied in hospitals. If a patient was found to self-injure, this behavior would be reported to the attending clinician, and additionally, the behavior would be addressed in therapy sessions or additional sessions would be administered as indicated. The staff were united in their attempts to deal with self-harm, even if they weren’t directly involved in patient care. To replace self-harming behaviors, methods for producing a pain stimulus were frequently adopted. These methods included putting ice, cold water, spices, or lemon on hands to heighten senses, snapping a rubber band around the wrist to provide pain stimulus, and drawing pretend wounds on themselves. Alternatively, one person had girls draw a beautiful butterfly on their hands so they could be reminded not to hurt the beautiful butterfly in case they had an urge to hurt themselves. Another approach was to draw a “hurt person” and describe what parts of this person were beautiful. The restrictions placed on the use of smartphones varied by department. During the inpatient period, one department strictly prohibited the use of any smart device. Other departments had time limits ranging from 30 minutes to 2 hours for phone use. One department did not monitor smartphone usage at all. The social workers served as a liaison between the family, the school, and the patients. The social worker would become involved with patient cases as needed to assist with social concerns, coordinate court documents, and plan postrelease treatment. Voluntary groups such as Big Brothers Big Sisters were proposed for connecting patients with elder mentors. While most social workers maintained a physical boundary with patients, one spent a significant amount of time with children. They even kept their door open most of the time so that patients could come to them with their concerns. Patients frequently confided in them, and while they sought to respect patients’ privacy, they occasionally informed those responsible for the treatment of vital information. However, clear boundaries were maintained outside of the workplace, and while patients occasionally wanted to form contacts on social media, they limited these connections to maintain their privacy.

### Insights from staff

The vast majority felt that the management of self-harm in inpatient settings is insufficient and that patients still find ways to injure themselves. When confronted with or dealing with patients’ self-harm, interviewees expressed a wide range of emotions. The emotions ranged from neutral and indifferent to extremely negative. Those who reported feeling indifferent rationalized their feelings by claiming that while self-harm is tragic, a professional often becomes accustomed to such situations and learns to stay impartial to effectively manage the circumstance and the patient. Others felt emotional during such circumstances but attempted to regulate their feelings to better handle such occurrences. Others reported feeling bad, sad, or distressed. According to an experienced clinician, such occurrences always feel “awful,” and the sentiment doesn’t ease with time. Perceived motives for why a child engages in self-harm also seemed to affect the emotional reaction of staff members. Additionally, most staff did not fear for their safety during work hours. However, there were incidents where patients injured staff members. Those working with adolescents felt more concerned for their safety. The general advice was to move in pairs and refrain from moving alone in the department. Also, most rooms were equipped with phones, so help could be called if needed. Staff were interviewed for ideas on how to improve dealing with self-harm. The common consensus was that eliminating self-harm instances may be unattainable. However, the focus should be on helping patients with emotion regulation, positive reinforcement, and devising alternative coping strategies. Not blaming the patient for their situation is essential. One person thought it might be useful if patients had a dedicated journal where they could express themselves and write down their feelings at different times. An increasing sense of autonomy was considered crucial. Also, the departments often had a mix of different patients, and such a mixture was considered to hinder treatment. There was hope that concentrating specific patient groups into a different department might prove beneficial as it allows tailoring and better management of disorders, especially among those not directly involved in patient treatment. While it was agreed that in-field experience improves observation methods and skillsets in dealing with self-harm, there was a wish for further training in addressing and understanding self-injurious behaviors and disorders associated with them. The majority of the nurses and some of the other staff felt there was a staff shortage and thought this affected how well children could be monitored and helped. One interviewee said they feel their job is “hard sometimes” (due to staffing problems, a low salary, and work pressure), but the supportive work environment and the need to help children make them stay. They also hoped there would be more support for healthcare workers. Other people said that their job is “emotionally demanding,” “draining,” and/or overwhelming. Not everyone thought staffing was a problem. Moreover, there was a notion that psychiatric disorders are stigmatized and that there is a lack of education regarding such problems. Thus, patients and families should be provided with proper information and education to improve understanding and awareness regarding mental health disorders and consequently change attitudes as an essential part of treatment. In cases of dysfunctional family dynamics, appropriate interventions should be in place, with social workers following up on the situation.

## Discussion

Self-harm seemed to be a prevalent issue among patients in clinical settings. Our study found that clinical management of self-injurious behavior included risk assessment, observation, communication between specialists, medication, counselling, removal of sharps, separation of patients with similar problems, and other methods, such as safety contracts, rewards or alternative stimuli that cause pain or mimic self-harm. Still, majority of staff did not consider inpatient management of self-harm to be sufficient.

Nonsuicidal self-injury was assessed upon admission through interviews, questionnaires, and an examination of the skin. We can also find a recommendation to use multiple assessment approaches (self-report, checklists, and structured and unstructured clinical interviews) in the literature [19]. Patient safety and monitoring were prioritized in all hospitals, with high-risk patients under stricter observation. Treatment plans were adjusted based on patient progress, and clinicians proactively discussed self-harm during sessions. In all hospitals, as part of the treatment, the monitoring, removal of sharp objects, and PRN medications were used. Such tactics are often referred to as standard treatment. However, it’s found that this current policy is often counterproductive [18].

NSSI monitoring is a major focus in many hospitals, and nurses play a vital role in this regard. Despite the high attention to self-harm monitoring, both in our study and in the literature, we found no strong evidence that it reduces the likelihood of self-harm. One study found no significant association between constant special monitoring and self-harm outcomes in acute psychiatric units. The researchers concluded that the lack of an association with self-harm suggests that self-harm can be reduced without risking patient safety [20]. The authors, who analyzed examples of nursing practices designed to maintain safety in psychiatric inpatient settings, such as close observations, seclusion, door locking, and defensive nursing practices, concluded that current nursing practices are not only ineffective but also harmful to both patients and nurses. Despite this, such strategies persist due to the validation provided by the expression of safety as a core value [21]. However, it’s crucial for nursing personnel to remain vigilant regarding alterations in the behavior or physical presentation of patients as these shifts could signal a change in mood. Such changes may indicate an elevated risk of self-harm. Recognizing these indicators has emerged as an important strategy for preventing suicide within inpatient care settings [18]. Some examples could include aggressive behavior, unauthorized attempts to leave the ward, and refusal of medication, which were common precursors to self-harm and suicide in inpatient units [22].

All departments in our study used some form of replacement strategy that either generated a pain stimulus or mimicked self-harming behavior to decrease or get rid of the urge to self-harm. While substitutes for self-harm are widely used, their use remains controversial [23]. Moreover, a considerable number of young individuals perceive harm minimization strategies as ineffective substitutes for self-harm [24]. Such strategies might be inefficient because painful stimuli may give reinforcement by boosting attention or relieving distress, resulting in a reinforcement loop and repeated self-harming behaviors [25]. Some departments used no-harm contracts with patients, making them promise not to harm themselves. However, there is no empirical evidence backing the efficacy of no-harm contracts for suicide prevention [26, 27], and little is known about its effectiveness for preventing self-harm.

The study revealed that individuals found alternative methods to harm themselves despite safety measures. Materials like pins, razors, and erasers were used, and during the COVID pandemic, metal strips from medical masks became common tools. The danger of alternative self-harm objects, such as face masks, was described in a case report detailing a patient’s injury caused by using the metal nose bridge [28]. Self-harm in adolescents occurred alone or in groups, and the most injured areas were the arms and thighs. Hospitals implemented strategies such as using plastic materials, restricting utensils, and closely monitoring patients during mealtime. Inpatient staff often attempted to remove the patient’s sharp objects, and visitors were instructed not to bring harmful items. However, these strategies may not be sufficient because patients still managed to conceal them, or as previously mentioned, found other ways to harm themselves.

Moreover, some studies indicate that the inpatient environment itself might contribute to self-harm. Psychological distress is a consistent factor for triggering self-injurious behaviors, and factors that can induce distress, such as a sense of isolation, feeling disconnected from others, and exposure to self-harm, can trigger the urge to self-harm among young people [9]. Furthermore, the inpatient environment itself, involuntary admission, and negative interactions with others are among other things that can cause distress in patients and induce self-harming behavior [29]. Important to mention, that staff interviewed in our study noticed that some children with no history of NSSI started self-harming in the hospital. Findings show that peer influence may play a significant role in adolescents’ involvement in NSSI. Research from longitudinal studies indicates that adolescents’ friends’ self-harm behaviors are linked over time with the adolescents’ own NSSI. Studies showed that friends’ self-harm significantly predicted NSSI, even after considering depressive symptoms. Gender emerged as a moderating factor, with friends’ behaviors predicting NSSI among girls but not boys [30]. In Lithuania, patients with nonsuicidal self-injurious behavior are mainly treated in outpatient settings. Children and adolescents with NSSI are often admitted to inpatient settings when they are at high risk of suicide, either by being referred by their treating psychiatrist or by coming directly to an emergency department.

Participants felt that patient care requires collaboration, and while it is not always simple, a supportive work atmosphere helps staff deal with patients who present with self-harm and other psychiatric problems. Social workers acted as liaisons between patients, families, and schools, offering support and planning postrelease treatment. Utilizing social services-based and integrated methods for post-self-harm care represents a vital step forward in suicide prevention efforts. Enhancing connections between social services and healthcare facilities to assist individuals seeking support after self-harm is recommended [31]. More training and protocols for dealing with self-harming behaviors were wished for. Raising public awareness regarding self-harm and psychiatric illnesses was viewed as a critical step in treating such problems, with some initiating a dialogue outside of the workplace within their inner circle.

Promising therapeutic approaches for NSSI encompass cognitive behavioral therapy, dialectical behavior therapy, and mentalization, alongside medications targeting the serotonergic, dopaminergic, and opioid systems [17]. A systematic review and narrative synthesis published in 2022 identified only seven articles confirming that nonrestrictive interventions to reduce self-harm among children in inpatient settings is an under-researched area. Among the seven described studies, three reported that DBT-based interventions resulted in significant reductions in rates of self-harm [32]. Still there is a lack of effective interventions for children and adolescents geared towards reducing NSSI [33]. One randomized-controlled trial evaluated the effectiveness of a brief, manualized family therapy in comparison to treatment-as-usual for reducing repeated self-harm leading to hospital attendance. The findings indicated no significant difference in the proportion of participants presenting to the hospital for repeated self-harm between the intervention group and the control group [34]. The meta-analysis examining the impact of interventions on self-injury and suicide among children and adolescents, and analyzing 112 randomized controlled trials, found that essentially almost all interventions did not significantly reduce self-harm. Furthermore, the results were largely consistent, regardless of intervention type, type of self-injurious, as well as the characteristics of the samples and studies. The authors concluded that existing specific interventions to self-injurious thoughts and behaviors (SITBs) may not effectively target the causal processes underlying SITBs due to the unknown causes, highlighting the critical need to identify and disrupt these processes to develop more effective interventions [16].

The experiences of various hospital staff we surveyed reflected that the measures to manage NSSI behavior are insufficient; specialists often experience various negative emotions regarding this issue. It was observed that most hospitals have systemic assessment and monitoring approaches, and in many hospitals, treatment aspects differed, ingredients of specific group interventions or individual therapies were not so clear. Effective management and treatment methods remain unknown after analyzing the practices and reviewing the literature. Although effective interventions are not so clear, it is known that self-harm decreases during adulthood [35]. Furthermore, the results of a recent qualitative study have refuted the view that less frequent self-harm is necessarily the most important criterion for assessing improvements in self-harm [36]. Our findings suggest that more research is needed to establish the efficacy of self-harm alternatives, such as substitution activities. Because individuals are shown to engage in self-harm for a variety of reasons [37], it is important to customize self-help strategies and interventions that take into account each individual’s unique characteristics, such as their personal circumstances, the specific triggers they encounter, their emotional state, and the level of distress they experience. We would like to agree with the conclusions of one study that the results emphasize the importance of interpersonal change in reducing or stopping self-harm. Although interpersonal factors are recognized as pivotal factors for self-harm, they frequently receive inadequate attention in self-management guidance and therapeutic interventions [38].

To our knowledge, this is the first study looking at self-harm approaches in hospitals in Lithuania. More qualitative and quantitative studies are needed to provide a comprehensive understanding of hospital-based approaches to self-harm and their effectiveness. Further research is necessary to enhance the understanding of the causes of self-harm, as well as to explore interventions aimed at addressing these causes.
